# Nucleation of protein crystals in pores and their growth

**DOI:** 10.1007/s12551-025-01347-1

**Published:** 2025-08-07

**Authors:** Christo N. Nanev, Emmanuel Saridakis, Naomi E. Chayen

**Affiliations:** 1 Rostislaw Kaischew Institute of Physical Chemistry, 1113 Sofia, Bulgaria; 2 Institute of Nanoscience and Nanotechnology, National Centre for Scientific Research “Demokritos”, Ag. Paraskevi, 15310 Athens, Greece; 3 Division of Systems Medicine, Department of Metabolism, Digestion and Reproduction, Faculty of Medicine, Imperial College London, London, W12 0NN UK

**Keywords:** Protein crystallization, Nucleation, Porous materials, Crystallization thermodynamics, Crystallization kinetics

## Abstract

X-ray diffraction enables determination of biomolecular structure but requires well-diffracting crystals that are notoriously difficult to grow. Porous materials can aid the crystallization of refractory proteins and, since knowledge of the mode of action of such materials may contribute to finding new crystallization inducers, this process has been studied thoroughly. It was established that, even under conditions where heterogeneous nucleation on flat surfaces is absent, a synergistic diffusion-adsorption effect inside a sufficiently narrow pore can increase the protein concentration to a level sufficient for crystal nucleation. The formation of a protein crystal in a pore begins with the assembly of molecules into a crystalline layer of monomolecular thickness, which is stabilized by its cohesion with the pore wall. We highlight thermodynamic considerations that provide an estimate of the importance of the protection due to the pore walls for crystal stability. In addition, molecular-kinetic considerations reveal further details of protein crystal nucleation assisted by porous materials. The observation that protein crystals nucleated by means of porous materials often display improved X-ray diffraction is of practical importance for structural studies. It is hoped that this review will guide scientists in their efforts to grow crystals of target proteins, complementing the usual trial-and-error strategies.

## Introduction

Therapeutic proteins in crystalline form are widely used to prevent and treat a wide range of diseases (Angkawinitwong et al. 2015). First, the crystallization process itself enables the removal of degraded, aggregated, or misfolded forms of proteins. Therefore, as an important purification step, protein crystallization provides opportunities to improve the quality of pharmaceuticals, enhance their safety and increase the efficiency of their production (Wang et al. 2018). Furthermore, the native configuration of proteins, which is of vital importance to maintaining their therapeutic function, is best preserved in crystalline drug formulations, which therefore allow for a much longer storage time. Third, a crystalline formulation is often key to suitable pharmacokinetic characteristics and controlled release. Homogeneity in crystal size and morphology is very important in this respect.

Furthermore, an in-depth knowledge of key details of protein molecular structures is needed to rationally design protein-based or protein-targeting drugs; a detailed knowledge of how ligands interact with binding sites at an atomic scale is indispensable. Nowadays, X-ray diffraction is the most widely used method for exploring such drug-binding sites, leading to the rapid optimization of drug–target interactions. Currently, X-ray diffraction accounts for about 90% of the structures deposited to the Protein Data Bank, while cryo-electron microscopy (cryo-EM) and, to a lesser extent, nuclear magnetic resonance complement X-ray crystallography rather than replacing it. Although cryo-EM made great strides in recent years, crystallography can still provide high-resolution structures over a wider range of proteins and protein–ligand complexes than any alternative method.

The notorious difficulty of growing 3D protein crystals suitable for X-ray diffraction studies is the main stumbling block in X-ray crystallography; after many decades of research, crystal growth largely remains a trial-and-error endeavour (Chayen and Saridakis 2008). Importantly, due to the lack of crystalline material to use for seeding, spontaneous crystallization is relied upon at the crucial early stages of the process. The latter begins with the formation of critically sized crystalline clusters, named nuclei, which are the smallest stable crystalline particles possible under the given conditions. To crystallize a protein, it is therefore necessary to induce the formation of such crystal nuclei, and this is not an easy task. The reason is that the attractive patches on the highly heterogeneous surface of protein molecules, on which mutual attachment of the crystal building blocks depends, vary depending on the composition of the solution. The crystallization of newly expressed proteins is therefore a tedious and uncertain process; it requires searching a large multi-dimensional parameter space to find one or a few sets of conditions suitable for the crystallization of the given protein.

In 2001, Chayen et al. (2001) introduced a new approach which uses porous materials as nucleation-inducing substrates (nucleants) to aid protein crystal nucleation. Since then, numerous experimental studies have confirmed that suitably engineered porous silicon, Bioglass, porous glass, porous gold, carbon nanotube meshes, synthetic zeolites and several other porous substrates, are effective in inducing protein crystal nucleation, e.g., (Rong et al. 2004; Saridakis and Chayen 2009, 2013; Khurshid et al. 2014; Asanithi et al. 2009; Kertis et al. 2012; Saridakis et al. 2011; Sugahara et al. 2008; Di Profio et al. 2009; Chayen et al. 2006; Eisenstein 2007; Stolyarova and Nemirovsky 2011). More recently, hydroxyapatite and titanium metal sponge were also used successfully in a quest to widen further the scope of porous nucleants (Nanev et al. 2021).

To explain these observations, it was argued that a combined diffusion-adsorption effect can increase the amount of protein inside sufficiently narrow pores to a level that is sufficient for crystal nucleation to occur, even under conditions where heterogeneous nucleation on flat surfaces is absent (Nanev et al. 2017). The reason is that diffusion is the only efficient mechanism of matter transport in confined spaces such as pores and sufficiently deep scratches; solution flow and convection are ineffective. Due to random walk with equal probability in all directions, once inside the pores the protein molecules reach their walls with a probability that is much greater than that of escaping back into the bulk of the solution. Provided there is some attraction between the protein molecule and the pore wall, some molecules will adsorb on it. Protein molecules stay in the adsorbed state for a sufficiently long time; thus, they are effectively trapped inside the confined pore space.

Of course, protein molecules also adsorb to and desorb from the surface of the crystallization vessel. However, the surface-to-volume ratio is incomparably greater for the pores than for the vessel; and provided the pore is sufficiently narrow, even after being desorbed, the confined protein molecules will be re-adsorbed with a probability which is much higher than the probability of re-adsorption of the protein molecules from the bulk solution (which is where the molecules are found after desorption from the vessel surface). Thus, when reiterated numerous times, this diffusion-adsorption scenario leads to gradual accumulation of protein molecules inside the pore.

The quantitative description of the dynamic adsorption–desorption process and diffusion-driven molecular displacement described above is simple: The departure of molecules from the shallow desorption potential well is described as an activated first-order rate process, e.g., see Atkins (1990). Accordingly, the desorption rate, *R*_d_ is written as:

1 $${R}_{\text{d }}= -\frac{\text{d}{c}_{\text{a}}}{\text{d}t}={k}_{\text{d}}{c}_{\text{a}}$$

which, after integration, gives:

2 $$c_a=c_a^0exp\left(-k_{d}t\right)$$

where *c*_a_ is the surface concentration of adsorbed molecules and *t* is time, *c*_a_^0^ being the initial concentration of adsorbed molecules.

The rate constant for desorption is ${k}_{\text{d}}= \theta \text{exp}(-{E}_{\text{d}}/{k}_{\text{B}}T)$ (Atkins 1990), where *E*_d_ is the desorption activation energy, *θ* an “attempt frequency” for desorption, *k*_B_ is Boltzmann’s constant and *T* the temperature.

The half-life time *τ*_1/2_ during which protein molecules are adsorbed at the pore wall is defined as the time when ${c}_{\text{a}}=\frac{{c}_{\text{a}}^{0}}{2}$. Thus:

3 $${\tau }_{1/2 }=\frac{\text{ln}2}{{k}_{\text{d}}} \sim ({E}_{\text{d}}/{k}_{\text{B}}\text{T})$$

It was found (Langdon et al. 2012) that the apparent energy barrier, *E*_d_ for desorption of an isolated protein molecule is in the range of 2–4 kJ/mol, which is less than 2*k*_B_T per molecule. Accordingly, it was concluded that “regardless of the protein identity or surface chemistry, the vast majority of individual protein objects exhibited [relatively] short residence times (< 1 s)” (Langdon et al. 2012). However, despite the relatively weak adsorption of the large protein molecules, they contact the surface at more sites than small molecules do, and therefore the adsorption capacity of protein molecules at solid surfaces is high even when the affinity between protein molecules and solid surfaces is very moderate (Dee et al. 2002).

Our estimate of the size of a pore that can accumulate protein because the adsorbed state is longer lived than the desorbed one, is based on the mean squared diffusion displacement $\overline{{x }^{2}}$, calculated by Einstein and Smoluchowski:

4 $$\overline{{x }^{2}}= 2\text{D}t$$

where D is the diffusion coefficient. Using Eq. (4), with the typical for proteins value of the diffusion coefficient D_prot_ = 10^−6^ cm^2^ s^−1^, it was calculated (Nanev et al. 2017) that a protein molecule can diffuse from one wall of a sufficiently narrow pore to the opposite wall, and adsorb on it, in less than 1 s. For instance, assuming time *t* = 0.5 s during which the protein molecules are in the desorbed state (i.e., half of the typical adsorption time of 1 s), Eq. (4) shows that a protein molecule can diffuse from wall to wall of a typical 10-μm sized pore, and adsorb there (the tacit assumption being that the molecule would follow the shortest path). And because in pores smaller than 10 μm the protein molecules are thus in the adsorbed state for longer than they are in the desorbed state, the concentration of protein in the solution inside the pore decreases; therefore, in the attempt to equalize it with the concentration in the bulk solution, diffusion brings new protein molecules into the pore. Furthermore, since Brownian motion is equally probable in all directions, the escape probability of a protein molecule from the pore is about 1/6. Therefore, provided the system is not too close to equilibrium, this synergistic diffusion-adsorption effect can increase the amount of protein inside pores narrower than about 1 μm, to a level that is sufficient for the onset of crystal nucleation. For a more accurate estimate, see the “Optimal pore width” section.

## Molecular-kinetic mechanism of protein crystal nucleation in pores

Scrutinizing the molecular-kinetic scenario of protein crystal nucleation in pores, the most likely mechanism by which crystals form has been proposed. It was realized (Nanev et al. 2021) that the formation of a three-dimensional (3D) protein crystal in a pore begins with the assembly of molecules in a crystalline layer of monomolecular thickness; two-dimensional (2D) crystal nuclei are preferred because they comprise fewer molecules than 3D crystal nuclei. The sudden accumulation of the (higher) number of molecules required for a 3D crystal nucleus would involve a much larger fluctuation than that needed for its 2D counterpart.

**Fig. 1:**
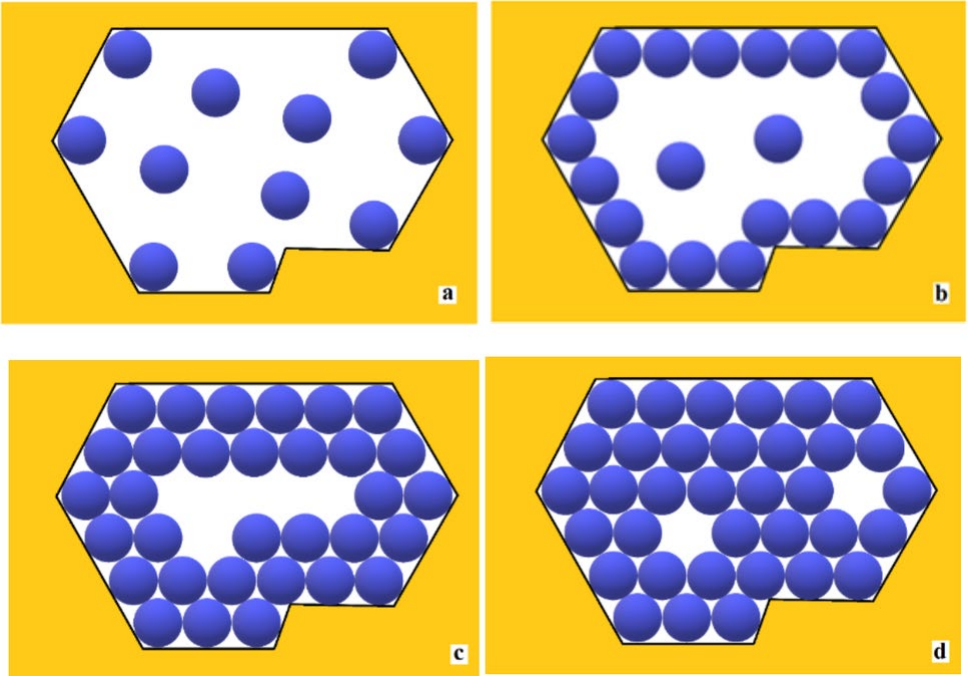
Top view of the gradual filling of a pore orifice. (a) Protein molecules are adsorbed (preferentially) at the re-entrant corners of the pore where they are bonded to two pore walls, while other molecules are floating in solution; (b) a necklace-like loop of adsorbed (on the inner surface of the pore orifice) protein molecules is the preliminary stage of protein crystal nucleation in the pore, while more molecules float in between (two molecules are shown in this figure); (c) a bilayer of adsorbed molecules is formed; (d) the pore orifice is filled due to multilayer adsorption, but some voids, the so-called vacancies, may remain in the 2D closest-packed crystal (Nanev et al. 2021)

The adsorption of protein molecules on the pore wall is an important prerequisite for the formation of 2D crystal nuclei (Nanev et al. 2021). A corresponding molecular-kinetic scenario of nuclei formation is reviewed here: Since protein molecules adsorb at statistically random sites on the pore wall, the formation of a “necklace” of protein molecules all at the same level in the pore (see Fig. 1a, b) is the necessary first step for the generation of a crystal nucleus. The molecules that are floating in between then bind to this “necklace”, thus forming the second, third and so on crystalline “necklaces” inside the first one (Fig. 1c, d). Of course, it is possible, especially with relatively wider pores, that the monolayer contains holes, see Fig. 1d. This, however, does not make the nucleus unstable; on the contrary, these vacancies in the layer increase its configurational entropy (see Supporting information to Nanev et al. 2021) and further stabilize the 2D crystal.

It should also be noted that, although the protein molecules diffuse deeper and deeper into the pore — reaching a mean squared diffusion displacement $\overline{{x }^{2}}$ in time *t* — and can adsorb at different depths inside it, the monomolecular protein layer is most likely to form somewhere near the pore orifice, where the molecules are most abundant. The main question, however, is at what size a protein crystal of monomolecular thickness formed in a pore is stable enough, i.e., expected to grow with negligible probability of dissolution.

The answer to this question is given from a thermodynamic point of view. By considering protein crystal nucleation in pores from this perspective, the destructive action of water molecules on the crystal periphery can also be estimated.

## Thermodynamic consideration of protein crystal nucleation in pores

As an introduction, the basic postulate of the thermodynamic description of crystal nucleation is recalled: The change in the Gibbs free energy *ΔG* required for formation of a crystal is the sum of two terms: (1) the free energy gain resulting from the transfer of molecules from the supersaturated mother phase into the crystal, which is proportional to its volume, the so-called ‘volume term’; and (2) the free energy penalty due to the formation of a new interface, a ‘surface term’, which is proportional to the total surface area of the crystal. When the crystal is very small, its surface-to-volume ratio is large, i.e., the surface term prevails, and such a crystal tends to disintegrate. However, increasing crystal size leads to the volume term increasing faster (to the power of three) than the surface term (to the power of two). The competition between these two terms thus determines the maximum total free energy change ΔG*, which is the energy barrier for the formation of the critical nucleus under isothermal and isobaric conditions; ΔG* also determines the edge length n* of the critical nucleus.

For a preliminary indication of the size of a protein crystal of monomolecular thickness formed in a pore, we compare it here with a rectangular 2D crystal floating like a raft on a solution (i.e., without contacting the pore walls). For simplicity, we assume that the latter is formed only from protein molecules located on the surface of the solution. (Since globular proteins are surface-active substances, this assumption is justified.) Importantly, although assembled in the 2D crystal, the protein molecules retain the same energetic interactions with the underlying solution as before their assembly, i.e., the free energy penalty due to the formation of the 2D crystal is only due to the creation of the crystal periphery. So, according to the above thermodynamic principle, the two terms which determine the change in the Gibbs thermodynamic potential $\Delta {G}_{2}$ with the change in the number *l* of protein molecules forming the edge of the rectangular raft-like 2D crystal are: (1) the number *l*^2^ of protein molecules in the 2D crystal (analogous to the ‘volume term’ in the 3D crystal); and (2) the new periphery (analogous to the ‘surface term’ in the 3D crystal). Thus:

5 $$\Delta {G}_{2}= - {l}^{2}\Delta \mu +4l\delta \kappa$$

where *Δμ* [erg] is the supersaturation and *δ* [cm] the edge length of the crystal building block, *κ* [erg/cm] being the specific periphery energy of the 2D crystal.

**Fig. 2:**
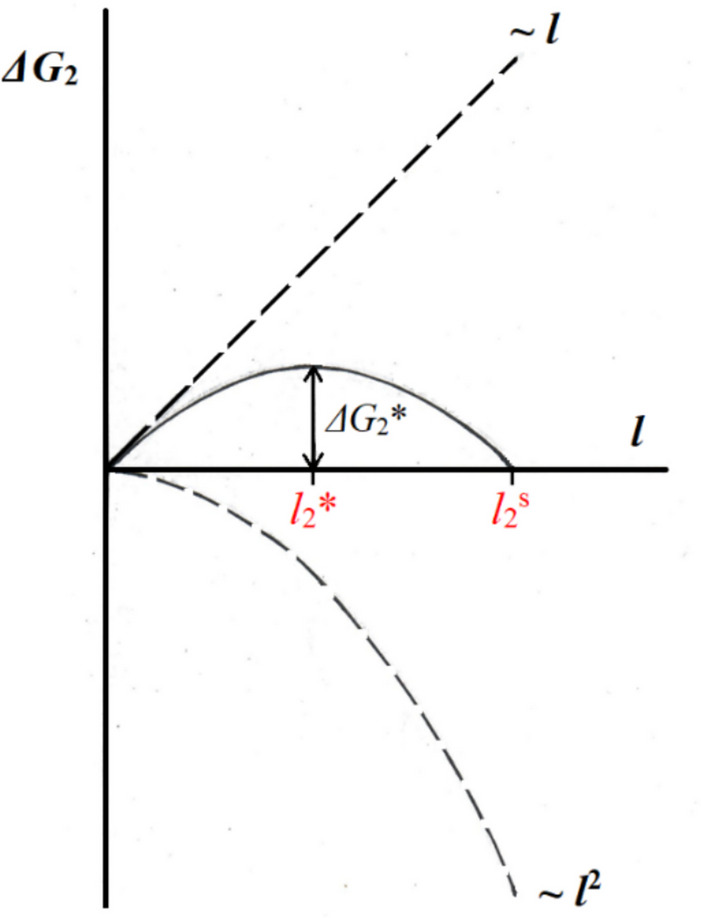
Change of the Gibbs free energy *ΔG*_2_ with the change in the number *l* of molecules forming the edge of the 2D crystal. *ΔG*_2_* determines the edge length ${l}_{2}^{*}$ of the critical nucleus, while *ΔG*_2_ = 0 determines the edge length ${l}_{2}^{s}$ of the fully stable supercritical nucleus, i.e., the 2D crystal which is destined to grow steadily (Nanev 2020)

As with the 3D crystal, when the 2D crystal is very small its periphery to ‘volume’ ratio is large, i.e., the periphery term (labelled ~ *l* in Fig. 2) dominates, and such a crystal tends to disintegrate. And again, increasing crystal size leads to the ‘volume’ term (labelled ~ *l*^2^ in Fig. 2) catching up fast. Thus, the competition between these two terms leads to the existence of a maximum of the Gibbs thermodynamic potential, which is the energy barrier for formation of the 2D critical nucleus; at constant supersaturation *Δμ*, the maximum *ΔG*_2_* gives the number ${l}_{2}^{*}$ of molecules forming the edge of the critical crystal nucleus floating like a raft on top of the solution:

6 $${l}_{2}^{*}=\frac{2\delta \kappa }{\Delta \mu }$$

Finally, the equilibration between the periphery and ‘volume’ terms gives the size of the fully stable 2D crystal, ${l}_{2}^{s}$ (see *ΔG*_2_ = 0 in Fig. 2).

The number ${l}_{2}^{s}$ of molecules in the fully stable 2D crystal is obtained from Eq. (5), setting $- {l}^{2}\Delta \mu$ and $4l\delta \kappa$ equal:

7 $${l}_{2}^{s}=\frac{4\delta \kappa }{\Delta \mu }$$

Keeping in mind that the critical crystal nucleus stands in unstable equilibrium with the supersaturated mother phase (meaning that the probabilities of dissolution and growth of this crystal are equal), the comparison with the completely stable 2D crystal floating like a raft on top of the solution with negligible dissolution probability, is more apt: From Eqs. (6) and (7), ${l}_{2}^{s}=2{l}_{2}^{*}$. The fully stable homogeneously formed 3D crystal nucleus is only one and a half times larger than the critical 3D crystal nucleus (Nanev 2020). The number, however, of crystal building blocks increases (due to the growth of the critical nuclei to completely stable ones) fourfold for the 2D crystal, while in the three-dimensional crystal the increase is 3.375-fold, i.e., there is no significant difference.

It is important for the present consideration that, since the periphery of any 2D crystal in the pore is protected from the destructive action of the water molecules, the value of *κ* in Eqs. (6) and (7) must approach zero; moreover, its effect is strongly reinforced by the attraction of the protein molecules to the pore wall, so that the 2D crystal nuclei in the pore can be very small. However, the synergistic effects of diffusion in the confined pore spaces and protein adsorption on the pore walls can increase the amount of protein inside the pores to a level that is just sufficient for crystal nucleation, while excessively high supersaturations are highly unlikely to be achieved. (A more detailed explanation of the effect of pore width is given in the “Optimal pore width” section.)

Although indirectly, the effectiveness of the protection by the pore walls of the periphery of the 2D protein crystal can be inferred from the destructive action exerted by water molecules on the periphery of the raft-like 2D crystal floating on the solution. To evaluate this destructive action, we compare the said 2D crystal with the completely stable 3D crystal nucleus (which is defined by the point of equilibrium between the crystal bonding energy $\Delta {G}_{\text{v}}$ and the destructive energy $\Delta {G}_{\text{s}}$, i.e., ${G}_{\text{v}}={G}_{\text{s}}$ (Nanev 2020)) which is completely immersed in the solution: Evidently, in the 2D case water molecules act destructively on the crystal periphery and on its lower surface, while water molecules act destructively on the entire surface of the 3D crystal. (For simplicity, the destructive action of water on the apexes and edges of the crystals is neglected.)

The convenient model of the so-called Kossel crystal (which is an idealized crystal built of tiny cubic building blocks held together by equal forces in a primitive cubic lattice) can be applied to this investigation. For the Kossel crystal, *ΔG* is:

8 $$\Delta G= - {n}^{3}\Delta \mu +6{\delta }^{2}{n}^{2}{\gamma }_{c}$$

where *n* is the number of the protein molecules forming the edge of the crystal, and $6{\delta }^{2}{n}^{2}$ the total surface area of the crystal; *γ*_c_ [erg/cm^2^] is its specific surface free energy.

The equilibration between the cohesive energy $\Delta {G}_{\text{v}}= {n}^{3}\Delta \mu$ and the destructive energy $\Delta {G}_{\text{s}}=6{\delta }^{2}{n}^{2}{\gamma }_{c}$ (i.e.,$\triangle G=0$ ) in Eq. (8) gives:

9 $$\Delta \mu =\frac{6{\delta }^{2}{\gamma }_{c}}{n}$$

And because, for the Kossel crystal, the specific surface free energy *γ*_c_ is proportional to the specific periphery energy *κ* [erg/cm] (Stranski and Kaischew 1934; Markov 2003):

10 $${\gamma }_{c}=\kappa /\delta$$

Equation (9) is transformed into:

11 $$\Delta \mu =\frac{6\delta \kappa }{n}$$

Comparison of Eq. (11) with Eq. (7) shows that a 50% higher supersaturation is required for complete stability of a 3D crystal nucleus completely immersed in solution, of the same edge length as a completely stable 2D crystal nucleus of monomolecular thickness floating like a raft on top of the solution. Evidently, this result can be explained by the more intensive destructive action of the water molecules on the 3D crystal and shows the importance of the protection of the protein crystals provided by the pore walls.

## Optimal pore width

Evidently, the wider the pore, the more unlikely it becomes to reach the supersaturation needed for crystal nucleation, while the narrower the pore, the easier it is to fill with enough protein for crystal nucleation. On the other hand, the pore opening is reached, and the protein molecules enter the pore, with the same probability with which they reach an equally large flat surface area, which means that smaller openings are less accessible. Therefore, for filling a pore with protein, the concentration of the protein solution is also important: the average distance between the protein molecules in the crystallizing solution can give an idea of the optimal pore width. Typically, protein crystals form in vitro at relatively high protein concentrations. For instance, concentrations of 50 to 60 mg/ml are commonly used for the crystallization of lysozyme. Rosenberger (1996) writes that “at the high concentrations typical of crystallization conditions, the average separation of protein molecules in the solution is comparable to their spacing in the crystal.” According to Fig. 1 in Fredericks et al. (1994), the average distance between neighboring molecules in solution is 8 nm. This would mean that, very roughly, 8 to 12 molecules will be present in the amount of solution that can fill a pore with a rectangular opening width of 100 × 100 nm. And by accumulating additional protein molecules via the diffusion-adsorption scenario, a pore of such width can potentially increase the amount of protein inside it to a level sufficient to promote crystal nucleation. Although this pore size is smaller than the 1 μm suggested initially, this estimate is consistent with the observation that trypsin and α-crustacyanin crystals grow from 0.3-μm pores of titanium metal sponge (Nanev et al. 2021). (Note that a 0.3-μm pore size is still much larger than the typical size of the crystal nucleus.)

However, the different shapes and sizes of protein molecules mean that they cannot all fit well into the same kind of pore: each protein needs a conformable pore shape and size. This explains why porous materials with a wide range of different pore sizes and shapes, such as Bioglass, porous silicon and nanoporous gold, are active nucleants for a wide range of protein crystals — but not zeolite, the pores of which are of equal sizes.

## Growth of protein crystals inside pores

**Fig. 3:**
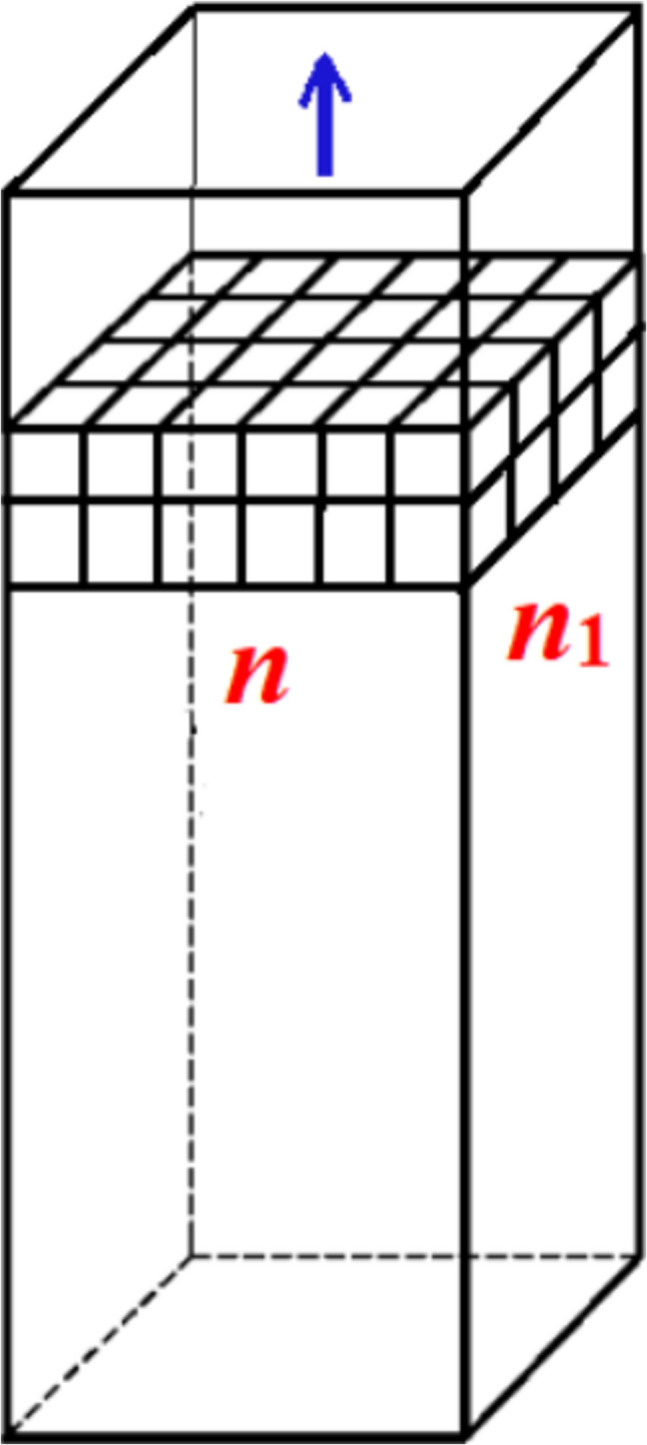
Double-layered Kossel crystal growing in a pore with the shape of a rectangular prism. The blue arrow shows the growth direction (Nanev et al. 2022)

Once the 2D protein crystal is formed in a pore, its further growth proceeds spontaneously, by the addition of new layers of monomolecular thickness. Using both Kossel and HCP crystal models, it was calculated that the deposition of a second protein layer of monomolecular thickness on the pre-existing first monolayer, a third layer on the second one, a fourth on the third, etc., proceeds with an increase in *ΔG*_v_ (Nanev et al. 2022), while the value of *ΔG*_s_ remains constant. We undertook to prove that, even without bonding of protein molecules to the pore walls, the strong bonding between successive monolayers is already sufficient for the spontaneous further growth of the crystal monolayer. We first considered a Kossel crystal with different numbers of molecules *n* and *n*_1_ at its edges (Fig. 3). We see that the bonding between the first and second crystal layers is secured by *nn*_1_ intermolecular bonds. Adding these to the (*n*—1)(*n*_1_—1) bonds (which are the same as in the first layer of monomolecular thickness), the increase in bonding strength is:

12 $$\frac{\left[n{n}_{1}+\left(n - 1\right)\left({n}_{1} - 1\right)\right]}{(n - 1)({n}_{1} - 1)}=1+ \frac{n{n}_{1}}{(n - 1)({n}_{1} - 1)}>2$$

i.e., the growing crystal becomes more than twice as strong. Of course, this calculation is merely indicative, since it does not take into account the bonding to the pore walls, which, as already shown, is very important.

Direct bonding to the pore wall is not, however, obligatory. For instance, in the closest packed crystal lattices, not all protein molecules interact directly with the walls of the pore. The reason for this is that the second layer of monomolecular thickness, deposited onto the fully stable hexagonal 2D crystal, is a ditrigonal layer, which is slightly smaller than the first layer. The lack of direct contact does not necessarily mean absence of any energetic interaction between the protein molecules at the edges of the successive crystal layers and the pore walls. It is plausible that there is an indirect bonding of these protein molecules, due to chains of water molecules forming through hydrogen bonds; such chains start from water molecules adsorbed on the pore wall and extend to hydrophilic amino acid residues located on the surfaces of the protein molecules that are incorporated into the crystal (see Discussion section). Moreover, the lack of direct contact is overcompensated by the substantially increased number of bonds with the underlying protein layers of monomolecular thickness: each molecule interacts with three molecules underneath it. Calculations using equations known from crystallography, have shown a significant increase in the stability of such crystals (Nanev et al. 2022).

Either way, being energetically favored, the deposition of layers of monomolecular thickness is an endless process, which, repeated many times, leads to the growth of a 3D crystal; there is thus no need to form a 3D protein crystal nucleus for the growth of a 3D protein crystal inside the pore.

## Growth of protein crystals outside pores

Because the volume of the protein crystal inside the pore is negligible compared to that of the whole crystal, the X-ray diffraction ability of a protein crystal is determined by the crystal grown outside the pore. Therefore, this growth stage is focused upon here.

Obviously, the point at which the protein crystal reaches the pore opening is a turning point in the growth process. Outside the pore, the protein crystal starts growing under completely different conditions — the protein concentration outside the pore is lower than inside it, and consequently the growth of the protein crystal must slow down. And the slower the crystal grows, the more perfect it is. The reason for this is that fewer impurities are incorporated by the slow-growing crystal, leading to fewer defects in its lattice, which means better diffraction quality (Saridakis and Chayen 2009). Therefore, by lowering the supersaturation at which the crystals nucleate and grow (compared to the supersaturation needed for conventional crystal nucleation), the use of porous materials contributes to the growth of high diffraction quality crystals for X-ray protein crystallography. Experiments (Asanithi et al. 2009) confirm this theoretical suggestion.

**Fig. 4:**
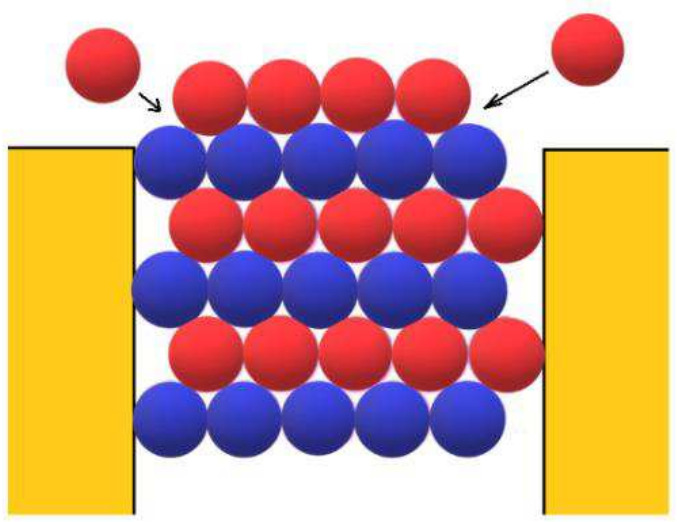
Cross-section of a pore shaped as a rectangular prism, with an HCP crystal just emerging from it. The alternating monolayers of the crystal are hexagonal A (blue spheres) and ditrigonal B (red spheres). For further growth outside the pore, protein molecules are attached to the crystal at the positions shown by the arrows (Nanev et al. 2022)

In addition, the open space outside the pore is advantageous for further growth of the 3D protein crystals, as they can be fed from more directions and expand laterally (see Fig. 4), which partially compensates for the reduced saturation — without removing the beneficial effect of the reduced inclusion of impurities on the quality of the growing crystal. Performing random walk, some protein molecules arrive at the crystal that is just protruding from the pore opening (see the two red spheres on either side of the opening, moving as shown by the arrows, in Fig. 4). Provided the supersaturation is not too low, such molecules can attach themselves to the crystal.

**Fig. 5:**
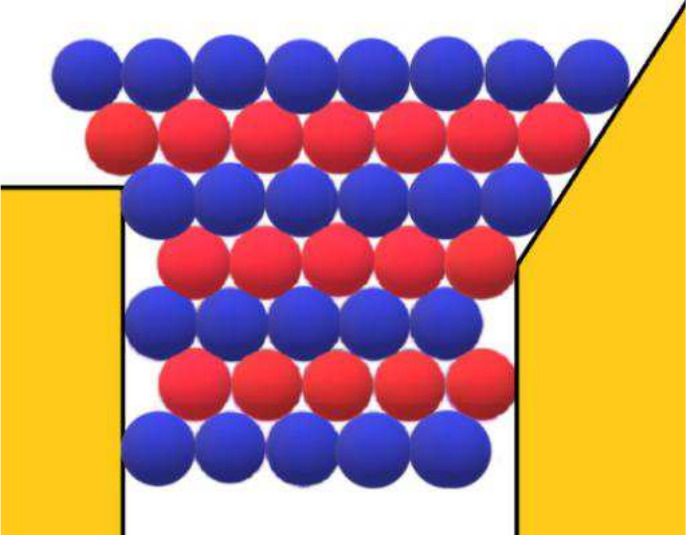
Growth of HCP crystal outside a pore with an inclined surface (shown on the right side of the pore), on which the molecules are more firmly bound. This is why an inclined surface facilitates the growth of subsequent crystal monolayers (Nanev et al. 2022)

However, the mere presence of open space outside the pore is by itself insufficient to promote the lateral growth of 3D protein crystals; the shape of the pore opening is also important. Sharply angular pore openings (as shown in Fig. 4) do not provide bonds with the pore wall for crystals emerging from the pore. Therefore, the red molecules in Fig. 4 are not bound sufficiently strongly to promote the lateral growth of the crystal outside the pore. But an inclined surface at the pore orifice (for example, like the one shown schematically in Fig. 5) can increase the number of bonds of the incoming molecules by providing additional bonds with the pore walls. Such pore openings are therefore more suitable for lateral growth of the crystal outside the pore. (Fortunately, real pores are generally devoid of sharp angles and have inclined surfaces at their openings.)

**Fig. 6:**
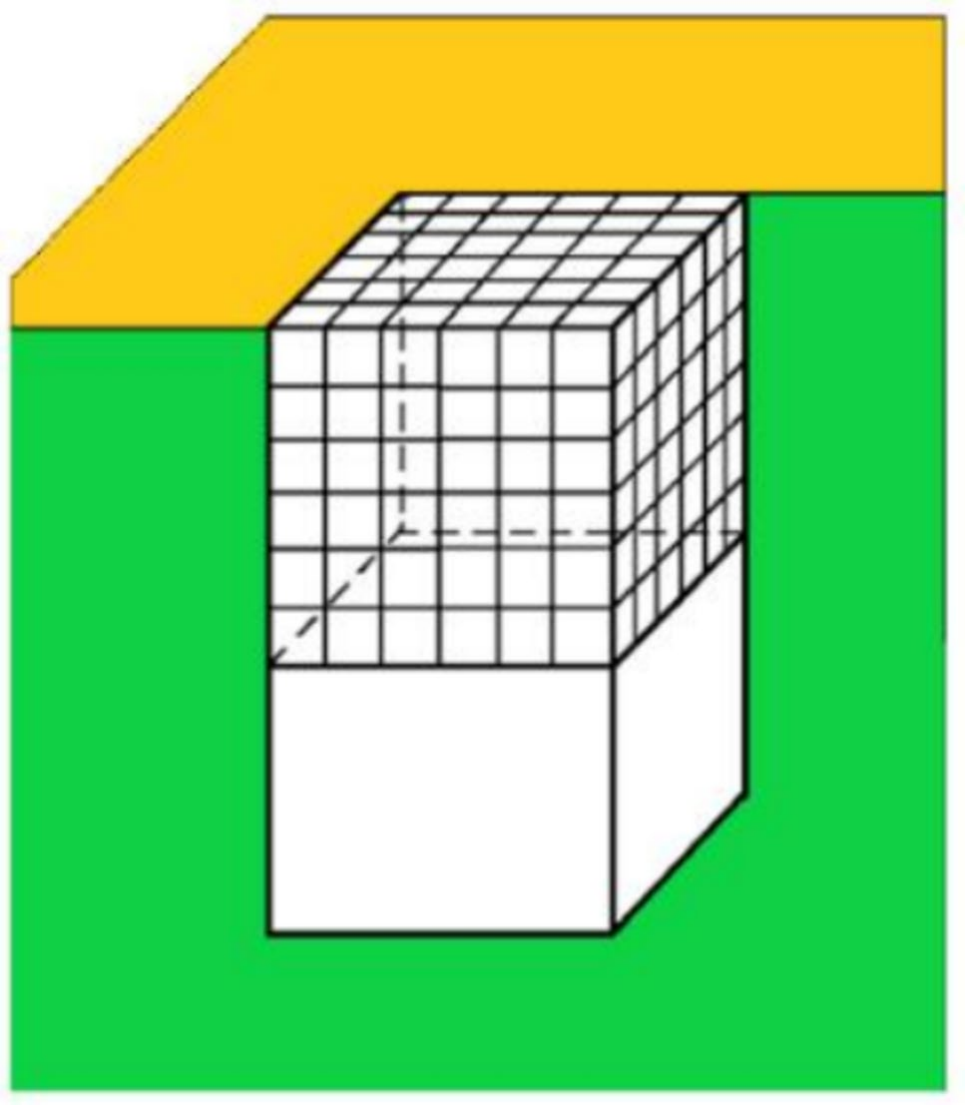
Cross-section (green) of a pore with a cubic crystal just reaching the pore opening. The surface of the porous material is in gold (Nanev et al. 2022)

Let us now scrutinize the conditions under which the protein crystal starts growing outside the pore. To this aim, we compare the protein crystal that has just reached the opening of the pore (see Fig. 6) with the completely stable homogeneously formed (at *ΔG* = 0) 3D crystal nucleus of the same volume, which is destined to grow steadily (Nanev 2020). Crystals inside pores can have diverse shapes, but the calculation of the value of the supersaturation needed for the growth of the crystal outside the pore is most easily done by considering the cubic (Kossel) crystal shown in Fig. 6. Being the only unprotected one, the face that has just reached the pore opening is vulnerable to the destructive action of the water molecules. Therefore, the supersaturation $\Delta {\mu }_{p}$ that is sufficient for its growth is obtained by transforming Eq. (9) into:

13 $$\Delta\mu_p=\frac{\delta^2\gamma_c}n$$

Comparing with the original Eq. (9), and as intuitively expected, the supersaturation ${\Delta \mu }_{\text{p}}$ is six times lower than the supersaturation $\Delta {\mu }_{\text{v}}$ at which the fully stable 3D crystal nucleus grows. Again, the addition of binding energies between successive protein layers of monomolecular thickness increases the binding energy *ΔG*_v_ of the protein crystal, so that *the same protein crystal continues to grow spontaneously outside the pore, without the need to form a new 3D protein crystal nucleus.*

**Fig. 7:**
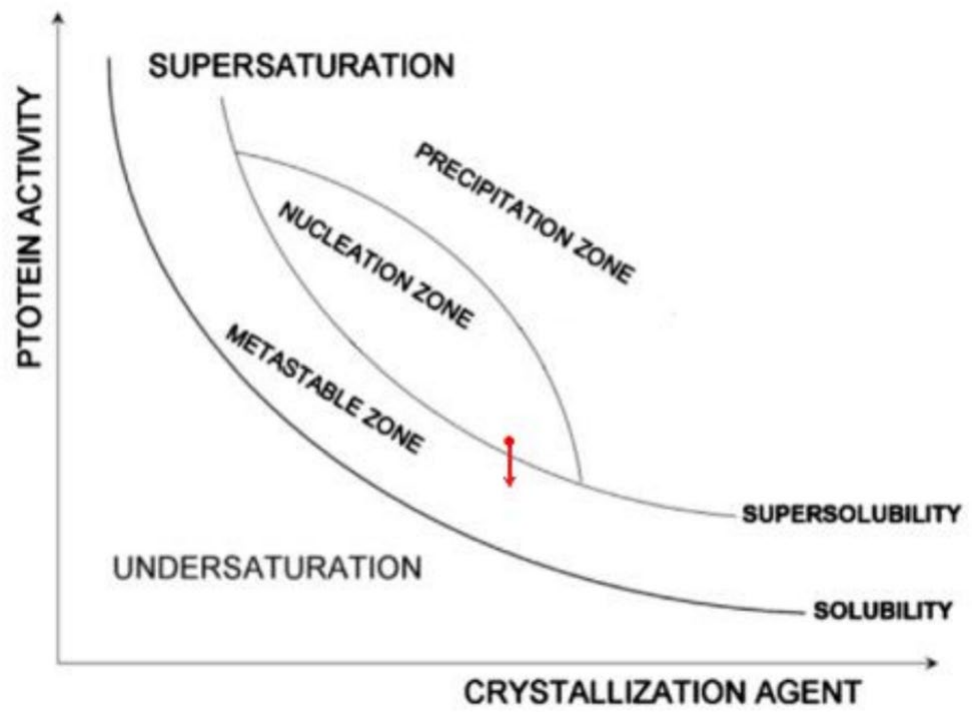
Ostwald-Miers phase diagram illustrating the nucleation of a protein crystal in a pore; the red point very close to the supersolubility curve shows the place in the nucleation zone where nucleation presumably occurs, while the 3D crystal grows most probably outside the pore in the metastable zone, as shown by the red arrow (Nanev et al. 2022)

Since protein crystals nucleate in pores at moderate supersaturations (achievable, as we saw, due to the synergistic diffusion–adsorption effect that arises from pore space confinement and interaction with the pore walls), we suggest that protein crystal nucleation in pores takes place in the nucleation zone, but very close to the supersolubility curve (see the red dot in Fig. 7). Consequently, we suppose it is most likely that $\Delta {\mu }_{p}$ (outside the pore) is below the supersolubility curve, i.e., already in the metastable zone (see the red arrow in Fig. 7).

## Experimental: diffraction quality of protein crystals grown by using porous materials

Since the earliest experiments on porous silicon (Chayen et al. 2001), many laboratories have developed and tested materials containing pores of different size distributions, but also other surface features promoting confinement, such as crevices, scratches, ‘nanowrinkles’ and other patterns. Some studies have only assessed nucleation induction times and rates, or overall number of ‘hits’ upon conditions screening, whereas others have also provided evidence of change in quality (size and/or diffraction quality) of the crystals grown with the help of porous materials. Most experimental work involved well-characterized model proteins, while a few worthwhile results were also obtained with more difficult to crystallize ‘targets’. The use of porous materials has expanded also to the crystallization of smaller molecules, such as medium-length 1-alcohols on mesoporous silicon (Henschel et al. 2008), aspirin on mesoporous silica (Diao et al. 2011; Juramy et al. 2025), or guanidino-γ-cyclodextrin on Bioglass (see below). Here, we will briefly describe some published results, focusing exclusively on porous materials (rather than those containing other protein-confining features), whose effectiveness appears to be due primarily to the existence of pores rather than to some specific chemical functionality of the surfaces.

Chayen et al. (2001) reported growth of large single crystals of four model proteins (hen egg-white lysozyme (HEWL), trypsin, catalase and thaumatin) and one target protein, a phycobiliprotein (C-phycocyanin, the structure of which had not yet been solved at the time and was later solved with crystals grown with the help of an alternative nucleation control method) from mesoporous silicon with a wide pore size distribution. This material was ineffective for the sixth protein tested, concanavalin A (conA).

Subsequently, Rong et al. (2004) reported crystallization of HEWL, thaumatin and apoferritin at metastable conditions using a commercially available porous glass of pore diameters 10–100 nm. The crystals were reported to be larger than those that could only be grown at higher supersaturations on standard glass.

The Chayen laboratory then developed a mesoporous bioactive gel-glass (Bioglass) specifically for protein crystal nucleation (2006), again with a wide pore size distribution (2–10 nm), that was effective at inducing nucleation at metastable conditions of all 7 tested proteins. These were standard model proteins HEWL, thaumatin and trypsin, and more difficult to crystallize proteins C-phycocyanin, lobster α-crustacyanin, α-actinin actin binding protein, and the C1 domain of myosin-binding protein-C (MyBP-C). Bioglass was also used to obtain the best-diffracting crystals of guanidino-γ-cyclodextrin (gguan), which is not a protein, but may be assimilated to a very small macromolecule with some protein-like properties stemming from its eight guanidino groups. Thanks to these crystals, its structure was solved to high resolution (Saridakis et al. 2022). Bioglass was also found to drastically reduce the nucleation induction time and to increase diffraction quality of crystals of proteins insulin glulisine (Li et al. 2019) and InHr2 (Nanev et al. 2022).

In 2012, Shah et al. (2012) used engineered mesoporous glasses of narrower pore size distributions that, as expected, showed specificity according to the size of each tested protein. Thus 3–4-nm pores were effective nucleants for thaumatin and trypsin, 4–6 nm for human serum albumin (HSA), 10–12 nm for conA and catalase, and 17–21 nm for ferritin.

Again in Chayen’s laboratory, Kertis et al. (2012) reported the use of nanoporous gold to obtain large single crystals of HEWL, thaumatin, trypsin and the more difficult to crystallize C1 domain of MyBP-C.

Guo et al. (2014), using porous polystyrene-divinylbenzene (SDB) microspheres as nucleation-inducing additives that adsorb protein molecules, obtained better diffracting crystals of conA, thaumatin, trichosanthin, heat-shock protein HSP90 and a kinase (ThiM).

Nanev et al. (2021) reported nucleation and growth at metastable conditions of crystals of trypsin, haemoglobin, a-lactalbumin, α-crustacyanin and glulisine, induced by porous hydroxyapatite.

Gerard et al. (2022) showed reduced nucleation induction time and increased nucleation rates of anti-CD20 monoclonal antibody, using mesoporous silica particles.

In the same year, two model proteins that had not been tested before with porous materials, α-chymotrypsin and ribonuclease A, were reported to yield larger and/or better-diffracting crystals when grown at metastable conditions in the presence of Bioglass, compared to crystals from the same samples grown at higher supersaturations (Nanev et al. 2022).

Another family of porous materials successfully tested for heterogeneous nucleation of protein crystals are carbon nanomaterials, such as Buckypaper (Asanithi et al. 2009), PEGylated carbon black, graphene oxide (Govada et al. 2016; Leese et al. 2016), PEGylated graphenes (Govada et al. 2022). Because of the added complexity of the specific surface chemistries and/or surface functionalization of these substrates, their performances will not be discussed here.

## Discussion

Protein crystal nucleation in pores is to some extent like capillary condensation of vapors — both phenomena are realized by multilayer adsorption. There are, however, many differences. Perhaps the biggest one is that while capillary condensation is due to an increased number of van der Waals interactions between molecules of the vapor phase within the confined space of a capillary, protein crystal nucleation in pores is due to much more complex interactions between protein molecules, *which, importantly, take place only under specific protein solution conditions*. Besides, in protein crystallization the adsorption of molecules on the pore walls is reversible and is followed by their diffusion when they are in a desorbed state; this is not the case for capillary condensation. Another difference between the two phenomena is that the surface tension of the liquid is very important for capillary condensation: Once a liquid phase is condensed, a meniscus immediately forms at the liquid–vapor interface — this allows equilibrium *below the saturation vapor pressure*; and the formation of the meniscus is a result of the surface tension of the liquid. In contrast, there is no liquid–vapor interface separating the liquid in the pore and in the bulk of the crystallizing system. Still another (obvious) difference between the two phenomena is that protein crystallization occurs in water, and water molecules play an important role during protein crystal nucleation and growth, including inside pores. The latter should not be neglected, but to the best of the authors’ knowledge, this role of water has not until now been considered; this is done below.

As already mentioned, see the “Growth of protein crystals inside pores” section, especially for closest packed crystal lattices, the protein molecules at the beginnings and ends of the successive crystal layers can be somewhat distant from the pore walls (see in Figs. 4 and 5). However, this distance does not imply the absence of any energetic interaction between them; such interaction can take place indirectly, through an intermediate monolayer of adsorbed water molecules and their H-bonds. In fact, recent studies by Maillet et al. (2022) using dynamic NMR relaxometry measurements, have shown that the thickness of the layer of water molecules adsorbed on porous Silica Glass (Vycor, Corning glass, code 7930) should be very close to the molecular diameter (i.e., 0.34 nm), which means that the adsorbed layer is composed of an almost uniform layer of molecules in contact with each other.

**Fig. 8:**
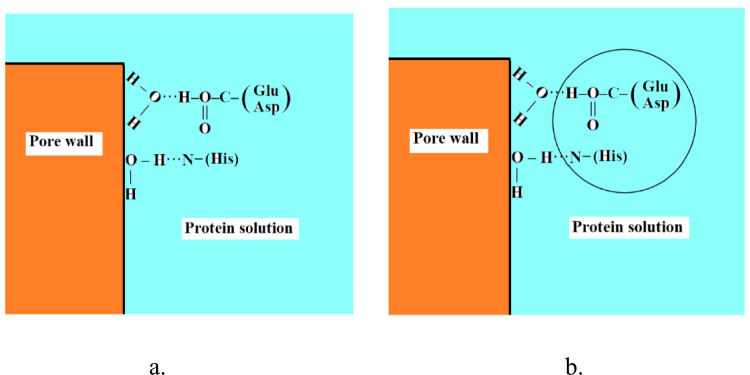
Two water molecules adsorbed on a pore wall. (a) The water molecules form H-bonds with two separate protein molecules, one having glutamic acid (Glu) and/or aspartic acid (Asp) amino acid residues (the latter being stronger), and the second having a Histidine (His) residue. (b) An alternative scenario involves one molecule (circled) with the same amino acid residues as in (a), forming H-bonds simultaneously with two adsorbed water molecules

It is therefore reasonable to assume that the adsorbed water molecules can form strong hydrogen-bonded assemblies (like bridges) with some amino acid residues of protein molecules. As already mentioned, such chains can start from water molecules adsorbed onto the pore wall and extend to reach the protein molecules incorporated into the crystal, see Fig. 8. Such bridges can for instance be readily formed by amino acid residues of glutamic acid and aspartic acid (which have carboxylic acid groups) and histidine (an essential amino acid that is present in most proteins and has a positively charged imidazole functional group) (Das and Baruah 2011). Usually, these amino acid residues do not participate in crystal lattice contacts (Dasgupta et al. 1997; Nanev 2008), and thus, the amino acid residues that do form the lattice contacts remain free to interact, enabling continuation of the crystal growth. (Note that some large biomolecules have several amino acid residues of glutamic acid and/or aspartic acid and/or histidine.)

## Data Availability

No datasets were generated or analysed during the current study.
